# Association between serum 25-hydroxyvitamin-D and Triglycerides-Glucose index among Indian adolescents

**DOI:** 10.1186/s40795-022-00568-x

**Published:** 2022-07-25

**Authors:** Akif Mustafa, Chander Shekhar

**Affiliations:** 1 International Institute for Population Sciences (IIPS), Mumbai, 400088 India; 2 Department of Fertility Studies, International Institute for Population Sciences (IIPS), Mumbai, India

**Keywords:** Vitamin D, 25 hydroxyvitamin D, TyG Index, Insulin resistance, India

## Abstract

**Background:**

Vitamin D deficiency has been found to associated with numerous skeletal and non-skeletal diseases including Diabetes Mellitus. Insulin Resistance (IR) is considered as one of the primary reasons of Type-2 Diabetes Mellitus (T2DM). The association between vitamin D deficiency and IR has been extensively explore in previous studies, but none of them focused on Indian adolescents, and none of them used the TyG index as IR marker. Hence, this population-based cross-sectional study investigates the relationship between insulin resistance (IR) assessed using the Triglycerides Glucose Index (TyG index) and vitamin D measured by serum 25-hydroxyvitamin-D (25(OH)D).

**Methods:**

For this study, we utilized data from the Comprehensive National Nutrition Survey (CNNS, 2016–18). The study is based on a sample size of 10,167 adolescents aged 10–19 years. The TyG index cut-off value of 4.65 was used to classify IR. We examined associations between the TyG index and serum 25(OH)D using multiple linear regression models adjusted for potential confounders. Odds of Insulin Resistance among vitamin D deficient/insufficient adolescents were assessed using multivariable logistic regression.

**Results:**

A significant negative association was found between serum 25(OH)D and the TyG index, where a 10% increase in serum 25(OH)D was associated with 0.56 (95% CI = -0.67, -0.45) unit decrement in the TyG index. The odds of having IR were 90% higher among vitamin D deficient adolescents (OR: 1.90; 95% CI = 1.62—2.23) compared to adolescents with adequate levels of vitamin D. The association between vitamin D deficiency and IR was independent of sex; in other words, the association between vitamin D and IR was significant in both the sexes.

**Conclusion:**

Independent of sex, this study found a significant inverse association between vitamin D and insulin resistance in Indian adolescents. The findings of this study highlight the utility of TyG index and the importance of vitamin D in lowering the risk of T2DM in future generations of the country.

## Introduction

Insulin resistance (IR) is a state of metabolic disorder in which the responsiveness of insulin-dependent tissues is reduced, leading to an elevated risk of diabetes mellitus and cardiovascular diseases (CVD). The insulin hormone helps in controlling the amount of glucose in the blood. When there is Insulin Resistance in the body, the target tissues start ignoring Insulin’s signal that is to get the glucose out of the bloodstream and put it into our cells. Resultantly, the entry of glucose into the cells becomes difficult, and it starts accumulating in the blood, which ultimately leads to chronic hyperinsulinemia [1]. Hyperinsulinemic-euglycemic clamp is the gold standard test for the assessment of IR; nevertheless, because of high associated cost, it is not commonly used. HOMA-IR (Homeostatic Model Assessment of Insulin Resistance) is the most widely used indicator for IR assessment, and it is calculated using plasma glucose and serum insulin levels. However, HOMA-IR is not viable for large-scale studies due to the relatively high cost of serum insulin estimation. Recently, numerous studies have suggested the Triglycerides and Glucose (TyG) index as an efficient marker for the assessment of IR [2–5]. The TyG index is a low-cost marker and easier to calculate as its calculation only requires serum triglycerides (TG) and fasting blood glucose levels.

Vitamin D deficiency is a serious public health concern all over the world. It is estimated that approximately more than one billion people worldwide had insufficient levels of vitamin D in 2007 [6]. In addition to its well-established role in musculoskeletal health (like osteoporosis, rheumatoid arthritis, fractures, bone metabolism etc.), low levels of serum 25-Hydroxyvitamin-D (25(OH)D) are also found to be associated with elevated risk of various non-skeletal disorders like type-2 diabetes mellitus (T2DM), cardiovascular diseases, infectious diseases, multiple sclerosis, depression, schizophrenia, obesity, and chronic obstructive pulmonary diseases (COPD) [7–16]. Vitamin D receptors are found in most of the body tissues, indicating that it has a wide range of clinical and physiological roles in the human body. [17].

The association between vitamin D deficiency and insulin resistance has been well-explored in previous studies. A one-year double-blind, randomized trial of 130 non-diabetic men aged 20–65 years found that the HOMA-IR index increased significantly in the control group while remaining steady in the group treated with vitamin D supplementation [18]. Furthermore, the positive effect of vitamin D on insulin sensitivity has been observed in a range of population subgroups, including pregnant women, diabetic patients, elderly population, adults, Asians, and a variety of other groups [19–23]. Although many studies have found a negative relationship between vitamin D and insulin sensitivity, other studies have shown no association or effect of vitamin D on insulin sensitivity. A systematic review and meta-analysis of 18 RCTs concluded that Vitamin D supplementation did not affect insulin sensitivity [24].

The TyG index is a novel index that has been found to be a potential risk marker for the development of IR, type-2 Diabetes Mellitus (T2DM), Metabolic Syndrome, and a variety of cardiovascular diseases [4, 5, 25, 26]. Several studies have been conducted to evaluate the ability of TyG index to predict IR. A study found a concordance of 0.91 between the TyG index and the Hyperinsulinemic-euglycemic clamp (gold standard test for IR diagnosis), indicating a strong synergy between the two tests [27]. Similar results were found in another study which showed that the TyG index diagnosed IR with a sensitivity of 95.6 and specificity of 79.9, and the study also reported that the TyG index had better predictive power than the HOMA-IR index [28].These findings suggest that the TyG index is an efficient marker of IR with lower cost and easier access compared to other IR markers such as HOMA-IR.

We observed two research gaps while doing the literature review: first, no study has examined the association between vitamin D and IR in Indian adolescents on national level. Second, most of the studies in this context have used the HOMA-IR, Matsuda index, Insulin sensitivity index (FIVGTT), or the Hyperinsulinemic-euglycemic clamp to assess IR; which implies that the association between vitamin D and TyG index has not been explored well enough. Furthermore, we noticed that the relationship between vitamin D and IR is inconsistent across the studies. Hence, this study, keeping the research gaps in mind, will explore the association between serum 25(OH)D and IR among Indian adolescents using the TyG index.

## Methodology

### Data

#### Data Collection 

The data from the Comprehensive National Nutrition Survey (CNNS, 2016–18) was utilized for this study. CNNS is a large-scale cross-sectional survey that provides robust data on nutritional status, anthropometric markers, food intake, and micronutrient levels of Indian children and adolescents aged 0 to 19 years where age 0 indicates those children who have not reached their first birthday on or before the day of data collection. The survey adopted a multi-stage, stratified, probability proportional to size cluster sampling design to collect data from 30 states of India. For data collection, the target population was classified into three strata: 0–4 years, 5–9 years, and 10–19 years. The total planned sample size was 1,22,100 children and adolescents from 2,035 primary sampling units. For the household survey and anthropometric measurements, a sample size of 40,700 individuals was planned in each of the three age groups, whereas for biomarker data collection, the planned sample size was 20,350 individuals in each of the age group. The response rate was 95% for individual interviews; however, for the biomarker data collection (blood sample collection), it was comparatively low (63–64%). Detailed information on household selection, questionnaire, data collection, instruments used, and data handling has been published elsewhere [29].

The CNNS collected data on a variety of parameters including socioeconomic and demographic characteristics of the households, morbidity history, anthropometric characteristics, micronutrient levels, anaemia and iron deficiency, and markers of non-communicable diseases. Data on food consumption, health education, and sanitation practices were also collected in the survey [29].

#### Blood sample collection

Parents and subjects were instructed regarding overnight fasting (8–10 h) before the sample collection. Trained phlebotomists obtained 10 mL of venous blood samples in the morning to assess micronutrient concentrations. The blood samples were transported to the nearest collection center in cold bags. There, the serum was separated and divided into aliquots within 6 h of sample collection. The aliquots were then stored at -20 °C until the analysis. Standard internal and external quality control and monitoring procedures were implemented for data collection [29]. Serum 25(OH)D concentration was measured with an antibody competitive immunoassay using the chemiluminescence (Siemens Centaur) method, serum triglycerides levels were assessed by spectrophotometry using the enzymatic endpoint method, and fasting plasma glucose was estimated by Spectrophotometry using the Hexokinase method. Serum cholesterol was assessed by spectrophotometry using ‘cholesterol oxidase esterase peroxidase’[29].

#### Study participants

**Fig. 1:**
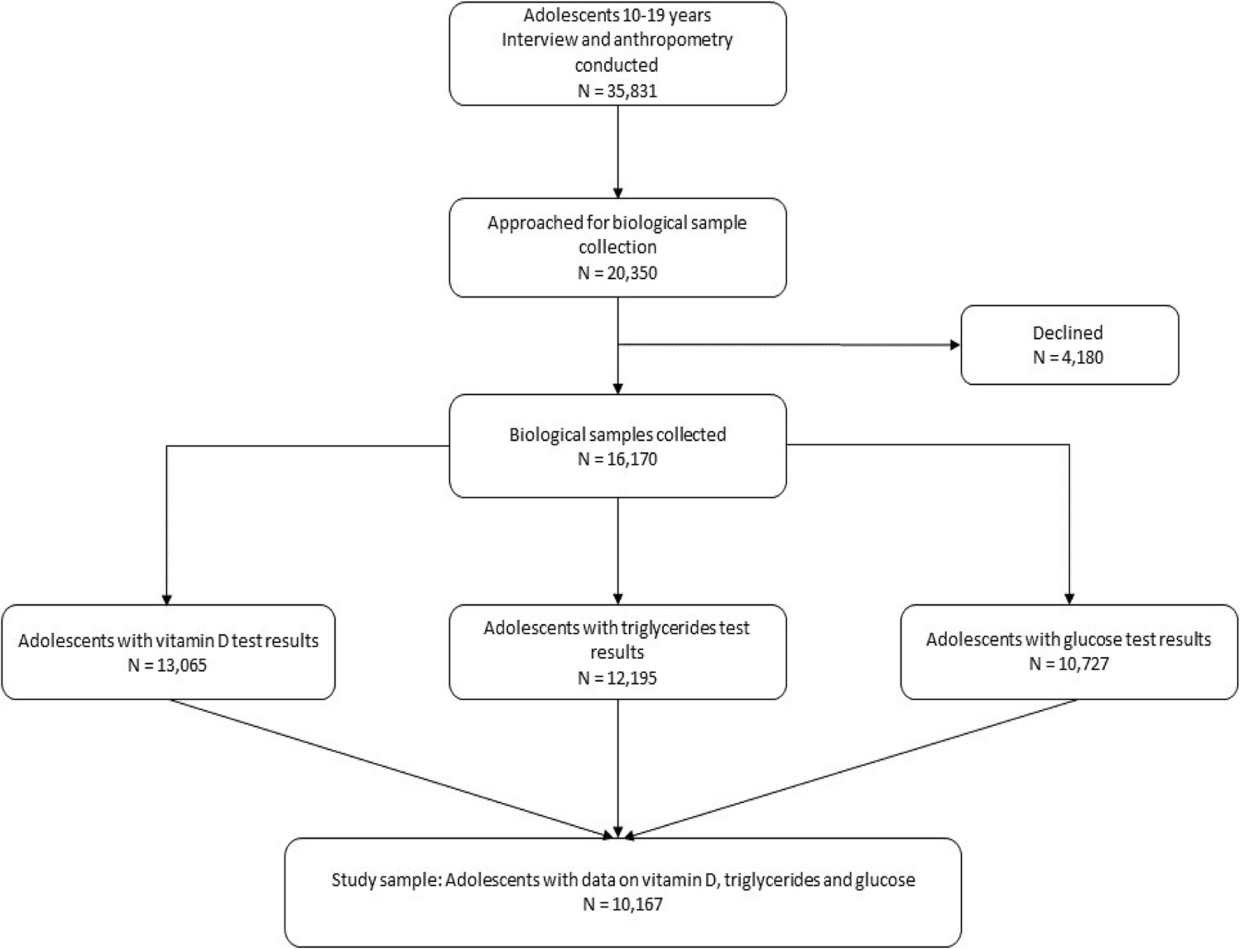
Flowchart of selection of study sample for analyses

Only adolescents aged 10–19 years were included in the analysis. Participants for whom data on vitamin D, fasting glucose, triglycerides, and BMI was missing were excluded from the analysis. The planned sample size was 20,350 individuals, but due to missing values, non-response, insufficient quality and invalid observations, the final sample size used in this study was reduced to 10,167. Flowchart of the selection of study participants for analyses is shown in Fig. 1.

### Variable description

#### Triglycerides-Glucose (TyG) index

The index was calculated as:

TyG index = Ln[fasting triglycerides (mg/dL) x fasting glucose (mg/dL)/2].

There is no fixed cut-off value of the TyG index for the assessment of IR; nevertheless, one thing is very clear: the higher the TyG index, the greater the risk of IR. For Indian population, there is no study that has identified the cut-off value of the TyG index for assessment of IR among adolescents; therefore, we adopted the cut-off value from a study conducted in Mexico, which found ‘4.65’ as the best cut-off value for the assessment of IR [30]. A binary variable for IR was generated as follows: “0” if TyG index < 4.65, and “1” if TyG index ≥ 4.65.

#### Vitamin D

Serum 25-hydroxyvitamin D (25(OH)D) is considered as the most reliable indicator of vitamin D levels in the human body [6]. Vitamin D deficiency was defined according to the criteria suggested by ‘Munns et al.’[31]. According to their criteria, vitamin D deficiency is defined as serum 25(OH)D level of < 12 ng/mL and insufficiency as 25(OH) D level between 12 and 20 ng/mL. 25(OH) D level higher than 20 ng/mL was accepted as adequate.

### Covariates

The association between the outcome variable and the primary independent variable was controlled for possible confounders and effect modifiers like age, gender, caste (Scheduled Caste(SC), Scheduled Tribe (ST), Other Backward Class (OBC), and Others), socioeconomic status (wealth index: Poorest, Poor, Middle, Rich, Richest), geographical region (North, Central, East, West, South, North-East), BMI, cholesterol level, serum creatinine, hypertension (Yes/No) and seasonal variations. Socioeconomic status was assessed using wealth index, which was provided in the dataset. The wealth index was created based on possession of common household items and facilities (like TV, bike, AC etc.). Detailed methodology of wealth index calculation is published elsewhere [32].

### Statistical analyses

Statistical analyses were conducted using STATA-16 and MS-Excel [33]. Relevant survey weights were applied for national representation of the estimates; however, weights were not employed in regression analysis. We created a scatter plot for graphical visualization of the association between 25(OH)D and the TyG index. To construct scatter plot, we divided the data into 40 quantiles with respect to serum 25(OH)D and calculated mean value of the TyG index and serum 25(OH)D in each quantile. Then we constructed a scatter plot by plotting mean TyG index against mean serum 25(OH)D; four-degree polynomial smoothening was additionally employed in the scatter plot. The association between serum 25(OH)D and the TyG index was assessed using Pearson’s product-moment correlation coefficient and multiple linear regression coefficients. Serum 25(OH)D was log-transformed as it had a right skewed-distribution. Three models were used for progressive degrees of adjustment to account for possible confounders. Model-1 was a bivariate model, that is the association between TyG index and vitamin D was not controlled for any covariate. Model-2 was adjusted for age, sex, caste, wealth index, geographical region, and seasonal variations (seasonal variation was controlled by including ‘month of interview’ variable in the regression). Model-3 was further adjusted for BMI, serum creatinine, serum cholesterol, and hypertension.

The odds of IR among vitamin D insufficient/deficient adolescents were assessed using multivariable logistic regression model. The model was adjusted for seasonal variations, demographic, socioeconomic and biological characteristics.

Because the data was hierarchical in nature, there was potential risk of intra-cluster correlation. To account for that, clustered robust standard error was used in the regression analysis (PSUs were set as clusters). Variance inflation factors (VIF) were estimated for each model to detect possible multicollinearity; however, no evidence of multicollinearity was found in the analysis.

## Results

The background characteristics of the study population are presented in Table 1. 51.4% of the respondents were males, and 48.6% were females. 24.6% of the adolescents were vitamin D deficient, and 36.7% were having insufficient levels of serum 25(OH)D. The mean value of the TyG index was found to be 4.48 ± 0.24. The value of the TyG index was higher among females (4.49) compared to males (4.46). The prevalence of insulin resistance was substantially higher among overweight/obese adolescents (37.9%). The TyG index varied significantly by geographical region (*p*-value 0.001), with the highest value (4.58) in the north-eastern region and the lowest value (4.41) in the southern region. The value of the TyG index was significantly higher among vitamin D deficient adolescents (4.51) compared to adolescents with adequate levels of vitamin D (4.43).

**Fig. 2:**
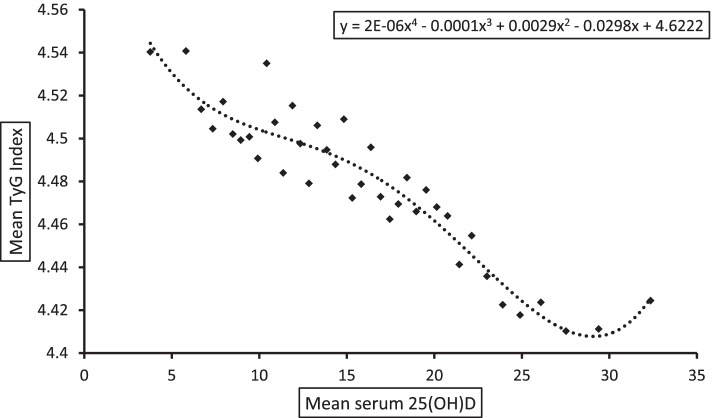
Scatter plot showing association between serum 25(OH)D and TyG index in adolescents of India (Comprehensive National Nutrition Survey, 2016–18), fitted with four-degree polynomial smooth

Figure 2 illustrates four-degree polynomial smooth and scatter plot of mean 25(OH)D and mean TyG index. The scatter plot clearly depicts a negative association between vitamin D and the TyG index; indirectly, it indicates that the risk of IR decreases with increase in vitamin D. We can also see that after a level of 23–24 ng/ml of 25(OH)D, the TyG index does not decrease any further. However, we did not have enough data points to comment on the association between the TyG index and 25(OH)D at higher levels of serum 25(OH)D [25(OH)D > 35 ng/ml].

A significant negative correlation (*r* = -0.14; *p*-value < 0.01) was found between TyG index and serum 25(OH)D. Table 2 illustrates the results of multiple linear regression analysis between serum 25(OH)D and the TyG index. Table 2 shows the results of multiple linear regression analysis. The results show a statistically significant negative association (*p*-value < 0.01) between serum 25(OH)D and the TyG index. According to Model-3, a 10% increase in serum 25(OH)D was associated with 0.56 (95% CI = -0.67, -0.45) unit decrement in the TyG index. Sex-specific regression results show that the association was significant in both the sexes; however, decrement in the TyG index with increase in serum25(OH)D was higher among males compared to females.

**Table 1:** Characteristics of study population, CNNS, 2016–18. Prevalence of Insulin IR is in percentage

Characteristics	Weighted %	Mean TyG Index ± SD	Prevalence of Insulin resistance
Gender**			
Male	51.40	4.46 ± 0.25	21.1
Female	48.60	4.49 ± 0.23	23.5
Age**			
10–14	53.75	4.49 ± 0.24	23.3
15–19	46.25	4.47 ± 0.24	21.1
Place of Residence**			
Rural	56.87	4.48 ± 0.24	21.4
Urban	43.13	4.47 ± 0.24	23.0
Wealth Index***			
Poorest	8.01	4.48 ± 0.24	22.8
Poor	13.07	4.49 ± 0.24	22.4
Middle	20.00	4.49 ± 0.24	24.0
Rich	26.83	4.49 ± 0.24	23.9
Richest	32.09	4.46 ± 0.24	19.8
Religion***			
Hindu	72.64	4.46 ± 0.24	20.4
Muslim	11.22	4.50 ± 0.24	24.8
Christian	10.13	4.54 ± 0.25	31.7
Sikh	2.50	4.43 ± 0.20	14.3
Other	3.52	4.54 ± 0.25	34.0
Caste***			
Scheduled Caste (SC)	20.93	4.45 ± 0.23	19.7
Scheduled Tribe (ST)	19.53	4.51 ± 0.25	26.9
Other Backward Class (OBC)	35.05	4.45 ± 0.22	17.3
Others	24.49	4.49 ± 0.24	23.3
Zone***			
North	21.97	4.45 ± 0.22	16.3
Central	10.17	4.42 ± 0.21	13.5
East	17.86	4.51 ± 0.24	26.6
West	10.33	4.42 ± 0.22	15.4
South	15.91	4.41 ± 0.23	14.2
North-East	23.77	4.58 ± 0.25	36.8
**BMI**^1^***			
Underweight	20.49	4.45 ± 0.23	17.7
Normal	71.14	4.47 ± 0.24	21.6
Overweight/obese	8.37	4.58 ± 0.25	37.9
Vitamin D***			
Adequate	38.65	4.43 ± 0.23	16.52
Insufficient	36.71	4.48 ± 0.24	23.56
Deficient	24.64	4.51 ± 0.24	26.02
**Hypertension**^2^**			
No	68.26	4.48 ± 0.24	22.2
Yes	31.74	4.49 ± 0.24	24.7
Serum Cholesterol (quantiles)***			
Q1 (113—118 mg/dl)	25.16	4.38 ± 0.22	10.22
Q2 (119—137 mg/dl)	25.86	4.44 ± 0.21	14.71
Q3 (138—158 mg/dl)	24.47	4.48 ± 0.21	19.17
Q4 (159—379 mg/dl)	24.51	4.60 ± 0.25	38.85
Serum creatinine (quantiles)***			
Q1 (0.10—0.50 mg/dl)	39.78	4.46 ± 0.22	14.52
Q2 (0.60—0.60 mg/dl)	21.05	4.45 ± 0.23	17.30
Q3 (0.70—0.70 mg/dl)	15.16	4.46 ± 0.23	17.90
Q4 (0.80—4.50 mg/dl)	24.02	4.54 ± 0.27	31.13
**Total**		4.48 ± 0.24	22.3

Note: These stars show the p-values of crude association between covariates and insulin resistance which are calculated using Chi-Square test

*SC* Scheduled Caste, *ST* Scheduled Tribe, *OBC* Other Backward Class

^1^BMI-Z score < -2SD = Underweight; -2SD ≥ Z ≤  + 1SD = Normal, Z >  + 1SD = Overweight/Obese

^2^Hypertension: systolic blood pressure > 139 mmHg or diastolic blood pressure > 89 mmHg. Q1, Q2, Q3, and Q4 are representing quantiles

^***^P-value < 0.001; **P-value < 0.01; *P-value < 0.05

**Table 2:** Results of multiple linear regression showing units change (95%CI) in the TyG index associated with a 10% increase in serum 25(OH)D among the Indian adolescents, CNNS, 2016–18

	Model-1		Model-2		Model-3	
	β	95% CI	β	95% CI	β	95% CI
Overall	-0.64***	(-0.73, -0.55)	-0.68***	(-0.81, -0.55)	-0.56***	(-0.67, -0.45)
Sex						
Male	-0.70***	(-0.84, 0.56)	-0.80***	(-0.97, -0.63)	-0.59***	(-0.75, -0.43)
Female	-0.52***	(-0.65, 0.40)	-0.63***	(-0.83, -0.43)	-0.55***	(-0.67, -0.43)

Note: Model-1: Showing crude association (which means not adjusted for any covariates). Model-2: Adjusted for age, sex, caste, wealth index, and geographical region. Model-3: Adjusted for Model-1 plus BMI, serum creatinine, serum cholesterol, and hypertension (Yes/No)

^***^p-value < 0.01. The analysis has been controlled for the seasonal variations

**Table 3:** Results of multivariable logistic regression analysis showing adjusted odds of IR among vitamin D insufficient and deficient adolescent, CNNS, 2016–18

	Overall	Gender	
		Male	Female
Vitamin D			
Adequate	1.00	1.00	1.00
Insufficient	1.38*** (1.97—1.59)	1.47*** (1.22—1.77)	1.25* (0.99—1.57)
Deficient	1.90*** (1.62—2.23)	1.92*** (1.54—2.41)	1.86*** (1.47—2.36)

Note: Odds ratios are adjusted for all the covariates as used in the Table 2, Model-3

The analysis has been controlled for the seasonal variations

^***^p-value < 0.01; **p-value < 0.05; *p-value < 0.1

Results of logistic regression analysis assessing odds of IR among vitamin D deficient adolescents are presented in Table 3. The results show that the adjusted odds of IR were 90% higher among vitamin D deficient adolescents (adjusted odds ratio (AOR): 1.90; 95% CI = 1.62—2.23) compared to adolescents with adequate levels of vitamin D. The odds of IR among vitamin D deficient adolescents were approximately similar for males (AOR: 1.92; 95% CI = 1.54—2.41) and females (AOR: 1.86; 95% CI = 1.47—2.36).

## Discussion

Based on a large-scale nationally-representative sample of general Indian adolescents, this study found a significant negative association between serum 25(OH)D and the TyG index, with the association being significant irrespective of sex. Consistent with the previous studies, this study also found a positive and statistically significant association between vitamin D deficiency and the risk of IR [22, 34].

This is the first population-based study in India to investigate the relationship between vitamin D and insulin resistance in Indian adolescents. Despite being a sunny country, vitamin D deficiency/insufficiency is significantly prevalent in the country. According to the CNNS, approximately 61% of Indian adolescents have insufficient vitamin D levels [29]. As cited in the previous studies, lifestyle factors and low dietary intake of vitamin D could be the possible reasons for high vitamin D deficiency in Indian adolescents [35, 36]. India is deemed as the diabetes capital of the world [37]. It is estimated that 77 million Indians have diabetes mellitus, and this number is projected to be 134 million in 2045 [38, 39]. Insulin resistance is one of the leading causes of T2DM [40]. As this study shows that vitamin D deficiency is significantly associated with IR among Indian adolescents, it emphasizes the importance of vitamin D in reducing the risk of T2DM in the upcoming generations of the country. We believe that the findings of this study have potential public health and policy implications.

A wealth of research shows that vitamin D supplementation reduces insulin resistance in vitamin D deficient individuals. However, less is known about how insulin sensitivity behaves at higher levels of vitamin D. Some of the previous studies show that the HOMA-IR index has the lowest value at the highest quantile of 25(OH)D [41–43]. On the other hand, a cross-sectional study conducted on 1887 individuals reported that serum 25(OH)D was inversely associated with HOMA-IR among individuals with serum 25(OH)D less than 30 ng/ml; but no association was detected among individuals with sufficient levels of serum 25(OH)D (30—100 ng/ml) [44]. One possible explanation of this lack of association could be that as vitamin D levels rise (> 30 ng/ml), insulin resistance decreases and stabilizes, resulting in no further decline in IR markers with increase in serum 25(OH)D; resultantly, the association between vitamin D and IR statistically diminishes. However, are very high vitamin D levels safe or beneficial for IR? This is a potential research question to be answered in future research.

Vitamin D receptors are found in almost all body tissues, including β-pancreatic-cells and insulin-responsive tissues such as adipose tissues, liver and skeletal tissues [45]. The 1α-Hydroxylase [1,25(OH)_2_D3] plays a crucial role in the activation of vitamin D synthesis. The results of various previous research have shown that the positive association of vitamin D with insulin sensitivity might be due to binding of 1,25(OH)_2_D3 to vitamin D receptors [46], activation of PPAR-δ [47], and induction of insulin receptors on target tissues [48]. 1,25(OH)_2_D3 interacts with Vitamin D Receptors in insulin-responsive cells; resultantly, it binds to retinoid X receptor (RXR). After that, the newly formed complex 1,25(OH)_2_D3-VDR-RXR connects with vitamin D receptors in insulin receptor genes. This leads to elevated transcriptional activation of Insulin receptor genes, which increases the number of insulin receptors in the cell [49]. This increased expression of the insulin receptor genes improves the insulin signaling pathway [50]. Thus, it seems that active vitamin D stimulates the expression of insulin receptors [50, 51].

Researchers have also proposed some indirect mechanisms of association between vitamin D and insulin sensitivity. Studies show that vitamin D elevates insulin sensitivity through calcium homeostasis and calcium influx through cell membranes [52]. As vitamin D enhances Ca^2+^ concentration in the cell, it increases the translocation of GLUT4 to the cell membrane in the target cell, which ultimately amplifies glucose uptake in the cell [53]. A recent study reported that vitamin D deficiency increases insulin resistance by provoking oxidative stress in hepatocytes [34]. The study proposed that when vitamin D depletes due to silencing of 1,25(OH)_2_D3 in L02 hepatocytes, it leads to significant production of reactive oxygen species (ROS) in the liver, which ultimately causes insulin resistance in the peripheral tissues [34, 54]. Although researchers have suggested various hypotheses and biological mechanisms to explain the association between vitamin D and insulin resistance; the underlying mechanism is still not well established.

This study has only focused on adolescents (10–19 years); future research should look into how the relationship between vitamin D and IR changes over the life course and in other demographic and socioeconomic groups. In earlier studies, the association between vitamin D and IR has been significantly inconsistent. The positive effect of vitamin D on insulin sensitivity have been observed in a variety of socioeconomic and demographic groups like Mexican children and adolescents [55], vitamin D deficient elderly [56], and pregnant females [21]. Other studies, on the other hand, found no relationship between vitamin D and IR among the elderly [23], Swiss adults [57], immigrants in the Netherlands [58], middle-aged T2DM patients [59], and numerous other groups [24, 60]. From the above pieces of evidence, it is clear that the positive association between vitamin D and insulin sensitivity is not universal. The researchers need to explore why some studies found a relationship between vitamin D and IR while others did not. It will also be interesting to see if the sample size has anything to do with the results of the studies; sometimes, observing the actual relationship or effect becomes statistically challenging when the sample size is too small.

A cross-sectional study conducted in Berlin (Germany) on 1887 individuals reported that vitamin D was inversely associated with insulin resistance among vitamin D deficient women but not in men [44]. In contrast, this study shows that the association between vitamin D and IR was independent of sex, indicating that the association was prevalent in both the sexes, although the strength of association was slightly stronger among males. Such differences in findings of the two studies could be attributed to differences in methodology and characteristics of the participants. The previous study’s participants were between the ages of 41 and 59 years and of Caucasian ethnicity, whereas this study is based on adolescents of Indian Asian race. Both the study populations are markedly distinct in terms of biological, socioeconomic, and lifestyle characteristics. Additionally, we used the TyG index to assess IR, whereas the above-mentioned study used HOMA-IR, making it more difficult to compare the two studies.

The present study has a few limitations. First, CNNS is a cross-sectional dataset; thus it was not possible to assess temporality, causality, and direction of association between serum 25(OH)D and insulin resistance. Second, there is no fixed cut-off value of the TyG index for the assessment of IR, due to which we had to borrow it from a research study conducted in Mexico. Another study limitation was the lack of data on critical vitamin D related factors such as skin color, time spent in sunlight, diet, and calcium levels. Recently, researchers have suggested to use free 25(OH)D for assessment of vitamin D levels instead of total 25(OH)D as it has been found that f25(OH)D reflect its biological actions better than t25(OH)D ^61^. In spite of these limitations, this study has several strengths. First of all, this study is a population-based study conducted on a nationally representative sample size. The CNNS used a robust sampling design and employed a rigorous internal and external quality control procedure during data collection. The data was collected throughout the year; hence, the association was adjusted for seasonal variation. We employed robust statistical methods to assess the relationship, and minimized all kind if biases like confounding, effect modification, intra-cluster correlation and multicollinearity.

In conclusion, this study found that serum 25(OH)D and the TyG index are significantly associated among Indian adolescents, independently of gender, indicating potential benefits of improving vitamin D levels for prevention from insulin resistance.

## Data Availability

The Ministry of Health and Family Welfare (MoHFW), Government of India, and Population Council (India) owns the CNNS data. Data are however available from the author upon reasonable request and with permission of Population Council (India).

## Abbreviations

TyG: Triglycerides and Glucose
IR: Insulin Resistance
25(OH)D: 25-Hydroxyvitamin-D
HOMA–IR: Homeostasis model assessment of insulin resistance
CNNS: Comprehensive National Nutrition Survey
ng/ml: Nanograms per milliliter
nmol/liter: Nanomoles per liter
